# Mixed-Phase MnO_2_/N-Containing Graphene Composites Applied as Electrode Active Materials for Flexible Asymmetric Solid-State Supercapacitors

**DOI:** 10.3390/nano8110924

**Published:** 2018-11-08

**Authors:** Hsin-Ya Chiu, Chun-Pei Cho

**Affiliations:** Department of Applied Materials and Optoelectronic Engineering, National Chi Nan University, Nantou 54561, Taiwan; s103328505@mail1.ncnu.edu.tw

**Keywords:** MnO_2_, N-containing graphene, composite, active material, specific capacitance, asymmetric supercapacitor

## Abstract

MnO_2_/N-containing graphene composites with various contents of Mn were fabricated and used as active materials for the electrodes of flexible solid-state asymmetric supercapacitors. By scanning electron microscopes (SEM), transmission electron microscope (TEM), energy-dispersive X-ray spectroscopy (EDS), X-ray photoelectron spectrometer (XPS), fourier-transform infrared spectroscopy (FTIR) and Raman spectra, the presence of MnO_2_ and N-containing graphene was verified. The MnO_2_ nanostructures decorated on the N-containing graphene were of α- and γ-mixed phases. N-containing graphene was found to reduce the charge transfer impedance in the high-frequency region at the electrode/electrolyte interface (R_CT_) due to its good conductivity. The co-existence of MnO_2_ and N-containing graphene led to a more reduced R_CT_ and improved charge transfer. Both the mass loading and content of Mn in an active material electrode were crucial. Excess Mn caused reduced contacts between the electrode and electrolyte ions, leading to increased R_CT_, and suppressed ionic diffusion. When the optimized mass loading and Mn content were used, the 3-NGM1 electrode exhibiting the smallest R_CT_ and a lower ionic diffusion impedance was obtained. It also showed a high specific capacitance of 638 F·g^−1^ by calculation from the cyclic voltammetry (CV) curves. The corresponding energy and power densities were 372.7 Wh·kg^−1^ and 4731.1 W·kg^−1^, respectively. The superior capacitance property arising from the synergistic effect of mixed-phase MnO_2_ and N-containing graphene had permitted the composites promising active materials for flexible solid-state asymmetric supercapacitors. Moreover, the increase of specific capacitance was found to be more significant by the pseudocapacitive MnO_2_ than N-containing graphene.

## 1. Introduction

The rapid development of portable and wearable consumer electronics results in the demand for high-performance energy-storage devices [1,2,3,4,5,6,7]. Solid-state supercapacitors (SSCs) have thus attracted growing attention, due to their reversibility, safe operation without the use of any liquid electrolyte, longer cycling stability than batteries, and higher power and energy densities than conventional capacitors [1,2,8,9]. Further improvement in energy density and searching for economic flexible current collectors are regarded as the two major challenges when pushing the technology of SSCs forward. Lots of effort have been made to investigate electrode active materials, to achieve higher specific capacitance. Carbonaceous materials such as activated carbon, carbon fibers, carbon nanotubes, graphene (G), and graphene oxides, etc., can be used as the active materials for electrodes of SSCs due to low cost, high conductivity, and chemical stability [10,11,12,13,14,15,16]. However, they usually show the drawback of lower specific capacitance, as compared to manganese oxide (MnO_2_) [17,18]. The theoretical specific capacitance of porous carbon materials is only 250 F·g^−1^ when the specific surface area is 1000 m^2^·g^−1^ [19]. Modification of pure carbon, like mixing activated carbon with conductive additives to obtain active materials with a high surface area and better contact for more efficient electrodes, was reported to improve the conductivity and capacitances of supercapacitors (SCs) [17]. Addition of heteroatom(s) or a pseudocapacitive material in G to fabricate composites was also conducive to the performance of SCs. Nitrogen (N) has a high electronegativity and lone pair of electrons, which can be in conjugation with the π electrons of G to enhance conductivity and electron transport. So, compared to pure G, N-containing G (NG) would be more favorable to the specific capacitance of SSCs.

Compared with carbonaceous materials, transition metal oxides and hydroxides (RuO_2_, MnO_2_, Ni(OH)_2_, Fe_2_O_3_, etc.) are deemed as more promising active materials for electrode applications to SSCs with large specific capacitances and high energy densities because the diverse oxidation states of the transition metals permit effective charge transfer [20,21,22,23,24]. Among them, MnO_2_ has attracted considerable attention due to its low cost, natural abundance, environmentally benign nature, and high theoretical specific capacitance (1370 F·g^−1^) [25,26,27]. Its pseudocapacitive characteristic can be attributed to the single electron transfer in the Mn^3+^/Mn^4+^ redox system [28]. Nevertheless, its poor conductivity and low ion diffusion constant may suppress the further progress [29,30]. The stability and potential application may be hindered by its problematic dissolution in electrolytes. To address these issues, the combination of MnO_2_ nanostructures with conductive carbonaceous materials to form hybrid structures has been adopted [31,32,33,34,35,36,37,38,39]. Some studies have focused on the development of MnO_2_-G composites, which took advantages of the high capacitance of MnO_2_ and the high conductivity of G simultaneously.

Despite the advantages of good electrochemical stability, high conductivity, and large specific surface area, G nanosheets tend to restack during the formation of solid materials due to the strong π-π interactions. The aggregation reduces the accessible surface area for adsorption and desorption of electrolyte ions, which would finally result in a small specific capacitance. The high conductivity and unique surface characteristics of the single-layer G nanosheets can be thereby lost [40]. Thus, suppression of the aggregation would greatly optimize the electrochemical properties of electrodes. One feasible method is to anchor transition metal oxides to the surface of G, working as spacers to separate adjacent G nanosheets. Transition metal oxides such as RuO_2_, NiO, CoO_x_, and MnO_2_ are appropriate candidates since they have been considered as extensively explored pseduocapacitor’s electrode materials showing high theoretical specific capacitance [41,42,43,44]. Introducing porous MnO_2_ nanostructures into G nanosheets would suppress the aggregation. The specific surface area of G was then increased, and more electrical conduction pathways were provided. The relatively higher gravimetric capacitances had been demonstrated for a variety of MnO_2_-G composites with low mass loadings, whereas some literature highlighted the importance of fabricating composites with higher mass loadings [45,46,47,48,49,50]. The optimization of mass loading would remain an important challenge.

The asymmetric SCs consisted of a carbonaceous negative electrode and a MnO_2_-based positive electrode offered enlarged operation voltage windows and thus improved power energy properties, compared with symmetric SCs [51]. For example, MnO_2_ nanostructures grown on activated carbon by a wet chemical reaction process was used as the positive electrode, which exhibited a high specific capacitance (345.1 F·g^−1^ at 0.5 A·g^−1^) and excellent cycle stability. It was assembled with an activated carbon negative electrode to fabricate asymmetric SCs, which showed high energy density of 31.0 Wh·kg^−1^ at a power density of 500.0 W·kg^−1^ [19]. Layered δ-MnO_2_ on N-doped G obtained by a hydrothermal approach was used as the cathode to improve the conductivity and present a high specific capacitance of about 305 F·g^−1^ at a scan rate of 5 mV·s^−1^. When it was assembled with an activated carbon anode using a gel electrolyte to fabricate flexible asymmetric SSCs (ASSCs), a maximum energy density of 3.5 mWh·cm^−3^ at a power density of 0.019 W·cm^−3^ was achieved [52]. Despite the progress, most MnO_2_-based SSCs did not exceed the energy density of lead acid batteries. Lots of efforts have been made to ASSCs with various electrode combinations [53,54,55]. Consequently, it is crucial to develop new active materials for more efficient electrodes applied to SCs.

In this study, MnO_2_/NG composites with various contents of Mn were fabricated by a hydrothermal approach and used as the electrode active materials for flexible ASSCs. Graphite paper on polyimide (PI) was employed as the soft substrate. NG can enhance the conductivity of composites and efficiently reduce the interfacial impedance. It can also serve as a better template for inducing the growth of MnO_2_ nanostructures than G. By the synergistic effect of MnO_2_ and NG, the specific capacitance, energy, and power densities were significantly improved. The MnO_2_ in the composites was found to be mixed phases containing γ-MnO_2_ and α-MnO_2_. The impacts of mass loading and the content of Mn on the capacitance parameters were also explored. The 3-NGM1 electrode with the most appropriate Mn content and mass loading of active material exhibited a high specific capacitance of 258 F·g^−1^ at a current density of 1 A·g^−1^. By calculating the cyclic voltammetry (CV) results, it had a superior specific capacitance of 638 F·g^−1^. The corresponding energy and power densities were 372.7 Wh·kg^−1^ and 4731.1 W·kg^−1^, respectively. The ongoing work regarding flexible ASSCs will be designed as using G as the negative electrode and a MnO_2_/NG composite as the positive electrode by employing a solid gel electrolyte. It is perceived that the optimized conditions of electrodes will lead to more enhanced capacitive behavior and cycle stability of the flexible ASSCs.

## 2. Experimental

### 2.1. Preparation of G

Graphite oxide (GO) was synthesized by the modified Hummers’ method using graphite powder [56]. A graphite oxidation procedure was executed before the synthesis of GO [57,58]. 4 g of graphite powder were added into a solution composed of 2 g of potassium persulfate (K_2_S_2_O_8_), 2 g of phosphorus pentoxide (P_2_O_5_), and 30 mL of conc. sulfuric acid (H_2_SO_4_). The mixture solution was heated to 80 °C under continuous stirring for 6 h. When it was cooled down to room temperature, rinse with deionized (DI) water was performed repeatedly by centrifugation until the neutral pH level was achieved. Afterward, 4 g of the pre-oxidized graphite powder were added into 100 mL of conc. H_2_SO_4_ solution in an ice bath. Then 12 g of potassium permanganate (KM_n_O_4_) were slowly added at 35 °C. The stirring was continued for 2 h until the color of the mixture turned to dark brown. Subsequently, a solution containing 200 mL of DI water and 40 mL of hydrogen peroxide (H_2_O_2_, 30 vol% in water) was added slowly while a violent chemical reaction occurred. A yellow-brown intermediate was produced when the reaction was completed, which was then put in a dilute aqueous hydrochloric acid (HCl) solution to remove metal ions. After ultrasonication for 1 h, rinse with DI water was repeatedly performed by centrifugation until the neutral pH level was achieved to obtain the GO powder.

20 mg of GO were added in 100 mL of DI water to prepare the GO solution. After ultrasonication for 2 h to make better dispersion of GO, the suspension was transferred to an autoclave, which was placed in a furnace for the hydrothermal process at 200 °C for 2 h. When cooling down to room temperature, the product was collected by filtration, and then dried at 80 °C for 12 h. After grinding, the powder of G was acquired [59].

### 2.2. Preparation of NG Composites

55 mg of G were mixed with 8.6 mL of ammonia hydroxide solution (28 vol%~30 vol%) in 70 mL of DI water. After ultrasonication for 2 h to make better dispersion of G, the suspension was transferred to an autoclave, which was placed in a furnace for the hydrothermal process at 140 °C for 6 h. When cooling down to room temperature, the product was repeatedly rinsed by DI water until the neutral pH level was achieved, and then collected by centrifugation. After dried at 80 °C for 12 h and grinding, the NG powder was obtained [52].

### 2.3. Preparation of NG/MnO_2_ (NGM) Composites

55 mg of G were mixed with 8.6 mL of ammonia hydroxide solution (28 vol%~30 vol%) in 70 mL of DI water. After ultrasonication for 2 h to make better dispersion of G, the suspension was transferred to an autoclave, which was placed in a furnace for the hydrothermal process at 140 °C for 6 h. When cooling down to the room temperature, five different weights of potassium permanganate (KMnO_4_) were added, respectively, to prepare the mixtures containing 8.9 mM, 17.8 mM, 26.7 mM, 35.6 mM, and 44.5 mM of KMnO_4_ solutions. After ultrasonication for 30 min, every mixture was transferred back to the autoclave, which was then placed in the furnace for another hydrothermal process at 160 °C for 2 h. When cooling down to room temperature, every mixture was taken out and repeatedly rinsed by DI water until the neutral pH level was achieved, and then collected by centrifugation. After dried at 80 °C for 12 h and grinding, five NGM composites with various contents of Mn were obtained [52]. They were named as x-NGM, in which x was 1, 2, 3, 4, and 5, respectively, to represent the five KMnO_4_ concentrations as mentioned above used during the preparation processes.

### 2.4. Fabrication of Electrodes

100 mg of G, NG and x-NGM composites were mixed with 12.5 mg of carbon black in 2 mL of absolute ethanol, respectively. After ultrasonication for 10 min to make better dispersion, 0.5 g of ethyl cellulose and 1 mL of terpineol were added to the three kinds of suspensions. Ultrasonication for another 10 min was performed to obtain more even mixing. The subsequent stirring for 10 min was to evaporate some ethanol, to achieve an appropriate consistency of the G, NG and x-NGM slurries for fabricating the electrodes of ASSCs.

A polyimide (PI) tape with the dimension of 3.5 cm × 2.5 cm was attached and stuck to a graphite paper to obtain a PI/graphite flexible substrate. On the other hand, a square hole with the length of 1.6 cm was made in the center of transparency, which was placed upper the flexible substrate and fixed by the 3M tape. The transparency was closely attached to the substrate, and the effective area was thus defined. Afterward, an appropriate amount of the G, NG and x-NGM slurries was uniformly coated within the square on the flexible substrate by the doctor-blade method. After standing at the room temperature overnight, the transparency was removed to acquire the electrodes, which were then calcined at 200 °C for 1 h to eliminate organics. Three different mass loadings were used for coating active materials on the PI/graphite flexible substrates. The resulting electrodes were named as Gy, NGy, and x-NGMy, in which y was 1, 2, and 3, to represent the mass loadings of 1 mg, 2 mg, and 3 mg, respectively.

### 2.5. Characterization

The surface morphologies of active materials were examined by field-emission gun scanning electron microscopes (SEM) (Hitachi, Tokyo, Japan and JEOL, Tokyo, Japan). Their microstructures and lattice fringes were examined by a high-resolution transmission electron microscope (HRTEM) (JEOL, Tokyo, Japan). The elemental mappings were obtained by the energy-dispersive X-ray spectroscopy (EDS). The chemical compositions of active materials were examined by the X-ray photoelectron spectrometer (XPS) (Thermo VG-Scientific, Waltham, MA, USA). According to the binding energies of photoelectrons emitted from the surface, the chemical state of each element could be ascertained. The vibrational modes of molecules identified from the absorption characteristics by Raman spectroscopies (Horiba Jobin Yvon, Paris, France) and infrared (Bruker, Billerica, MA, USA) ranging from 400 cm^−1^ to 2000 cm^−1^ and 400 cm^−1^ to 4000 cm^−1^, respectively, were used to determine the chemical compositions, bond configurations, and molecular structures of the active materials.

### 2.6. Electrochemical Measurements

The electrochemical properties were characterized by CV, galvanostatic charge/discharge (GCD), and electrochemical impedance spectroscopy (EIS), using a potentiostat/galvanostat (CH Instruments, Austin, TX, USA) as the analyzer. When the measurements on the electrodes coated with active materials were performed, a 5 M LiCl solution was employed as the electrolyte, and a three-electrode configuration consisting of a platinum (Pt) wire as the auxiliary electrode and silver chloride (Ag/AgCl) reference electrode was adopted.

The capacitance characteristics of the electrodes could be determined by the areas inside the CV curves obtained at different scan rates and the symmetry of the GCD curves obtained by different current densities. By Equations (1) and (2), the gravimetric specific capacitances C_CV_ and C_C-DC_ of individual electrodes were calculated from the CV and GCD curves, respectively [60]:

(1) $$C_{\mathrm{CV}}=k\frac{\int i}{m\cdot s}$$

(2) $$C_{C-\mathrm{DC}}=k\frac{i\cdot \Delta t}{\Delta V\cdot m}$$

(3) $$E_{\mathrm{EL}}\left(\mathrm{Wh}/\mathrm{kg}\right)=\left(\frac{1}{4}\times C_{\mathrm{CV}}\times V^{2}\right)/3.6$$

(4) $$E_{\mathrm{EL}}\left(\mathrm{Wh}/\mathrm{kg}\right)=\left(\frac{1}{4}\times C_{C-\mathrm{DC}}\times \Delta V^{2}\right)/3.6$$

(5) $$P_{\mathrm{EL}}\left(W/\mathrm{kg}\right)=E_{\mathrm{EL}}/\left(\Delta t\right)$$

where k is the electrode constant (usually 2 for a single electrode and 4 for a couple of electrodes), i is the discharging current, ∫i is the integral area of a CV curve, m is the mass of electrode active materials, s is the scan rate (100 mV·s^−1^ in this work), ∆t is the discharging time, and ∆V is the potential window subtracting the initial potential drop. In this study, the potential windows for individual electrodes were −1.9 V to 1.0 V. After substituting C_CV_ into Equation (3) and C_C-DC_ into Equation (4), the energy densities of the electrodes (E_EL_) could be calculated. By further substituting E_EL_ into Equation (5), the power densities of the electrodes (P_EL_) were obtained [60]. On the other hand, the electronic and ionic transports across the interface of active material in the electrodes were investigated by EIS. The frequency range for EIS was 10^−2^ Hz to 10^5^ Hz. The AC amplitude was set as 10 mV between two electrodes.

## 3. Results and Discussion

**Figure 1:**
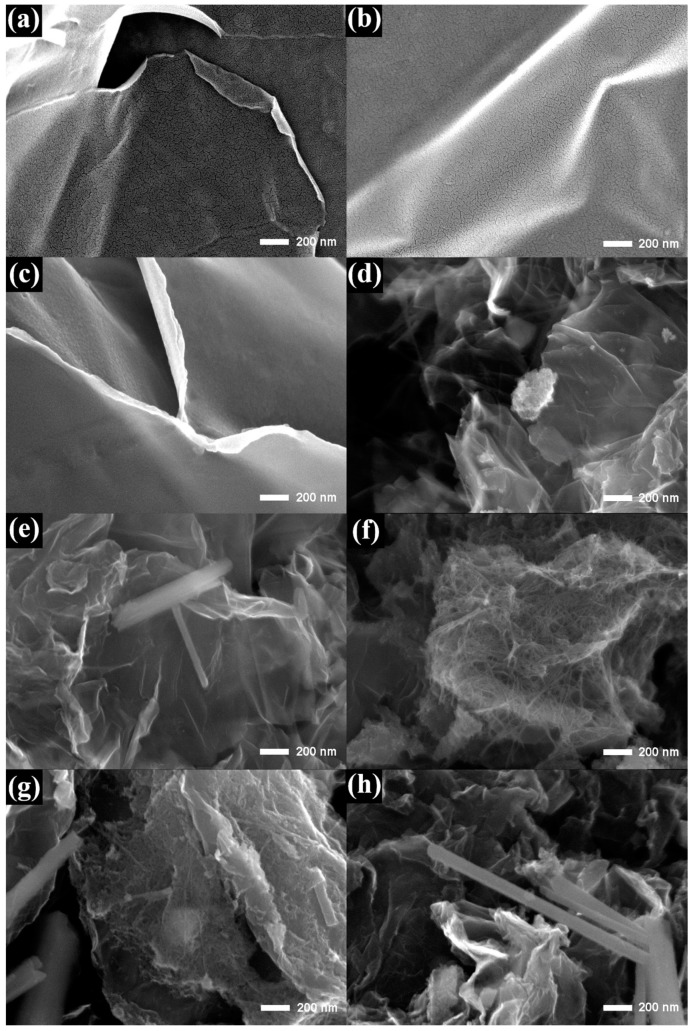
SEM micrographs of (**a**) graphite oxide, (**b**) G, (**c**) NG, (**d**) 1-NGM, (**e**) 2-NGM, (**f**) 3-NGM, (**g**) 4-NGM, and (**h**) 5-NGM.

**Figure 2:**
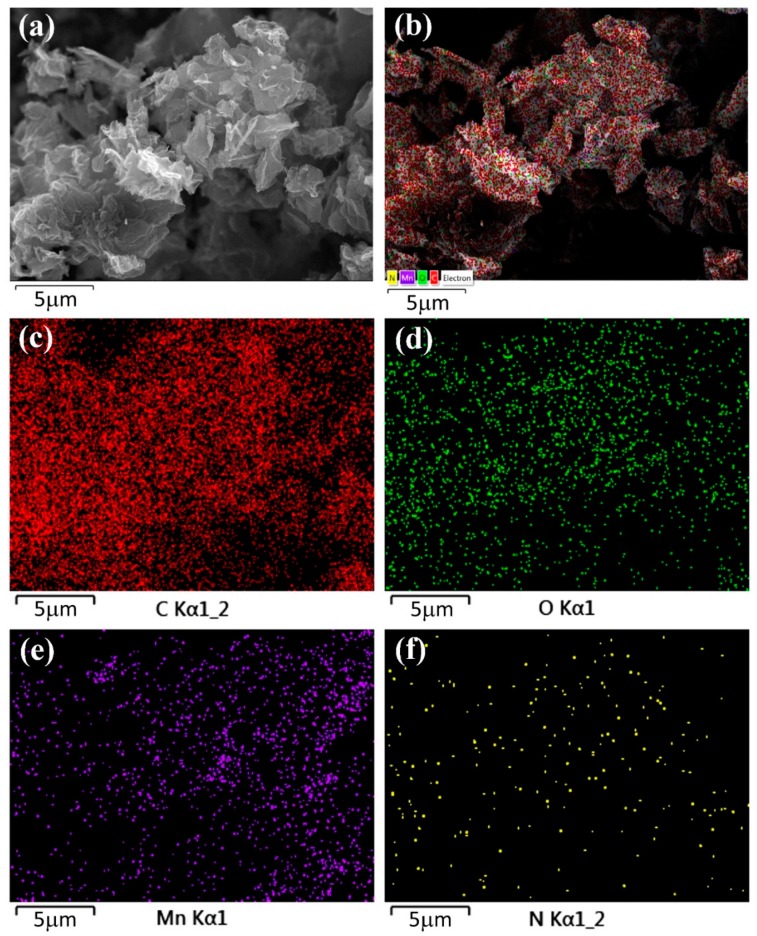
(**a**) SEM and (**b**) EDS layered images of 2-NGM. Elemental mappings of 2-NGM: (**c**) C, (**d**) O, (**e**) Mn, and (**f**) N.

The surface morphologies of graphite oxide, G, NG, and x-NGM composites at different magnifications were examined by SEM. The ultrathin sheet-like structures of graphite oxide, G, and NG were observed, as displayed in Figure 1. There are fine needle structures in x-NGM, which grow on the surface of NG, as shown in Figure 1d–f. The changes in the content of Mn were discovered to impact the morphologies and structures of x-NGM composites significantly. When the concentration of KMnO_4_ used during the preparation was higher to increase the content of Mn, a larger dimension of the needle structure resulted, as shown in Figure 1e,f. When the concentration of KMnO_4_ was 26.7 mM to fabricate 3-NGM, the intertwined MnO_2_ nanowires formed. When the KMnO_4_ concentration increased to 35.6 mM and 44.5 mM to obtain 4-NGM and 5-NGM, the MnO_2_ nanorods were observed, as displayed in Figure 1g,h. For further confirmation, elemental mappings were examined. Figure 2 demonstrates the presence of the C, O, N and Mn elements in 2-NGM and their even distributions. It verifies the successful preparation of the x-NGM composites as well. The sparser distribution of the N element was attributed to the relatively lower proportion of the N content in the composite.

**Figure 3:**
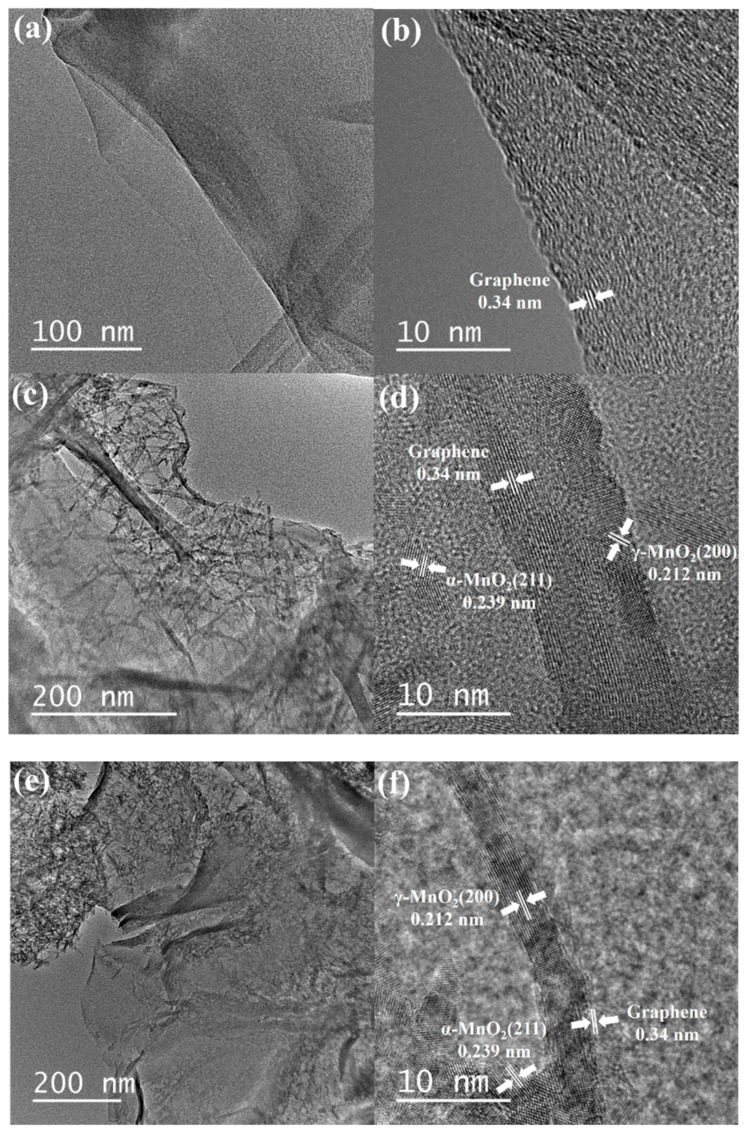
TEM micrographs and microstructures of (**a**,**b**) NG, (**c**,**d**) 2-NGM, and (**e**,**f**) 3-NGM.

Figure 3 displays the TEM micrographs and microstructures of NG, 2-NGM, and 3-NGM. The semitransparent membranous structure can be observed, as shown in Figure 3a,c,e, demonstrating the existence of NG in the composites. The lattice fringe spacing value of 0.34 nm corresponds to the d-spacing of G crystalline plane, indicating the successful formations of G and NG by the hydrothermal method [61]. Lots of fine needle structures appear on the NG surfaces of 2-NGM and 3-NGM, as shown in Figure 3c,e, respectively. The needle structure in 2-NGM is larger than that in 3-NGM. By the high-resolution atomic images in Figure 3d,f, the MnO_2_ in the x-NGM composites is discovered to be a two-phase mixture. Namely, the co-existence of γ-MnO_2_ and α-MnO_2_. Another two lattice fringe spacing values of 0.212 nm and 0.239 nm correspond to the (200) plane of γ-MnO_2_ and (211) plane of α-MnO_2_, respectively [29], which also contribute to confirming the presence of MnO_2_ and the successful preparation of the x-NGM composites.

**Figure 4:**
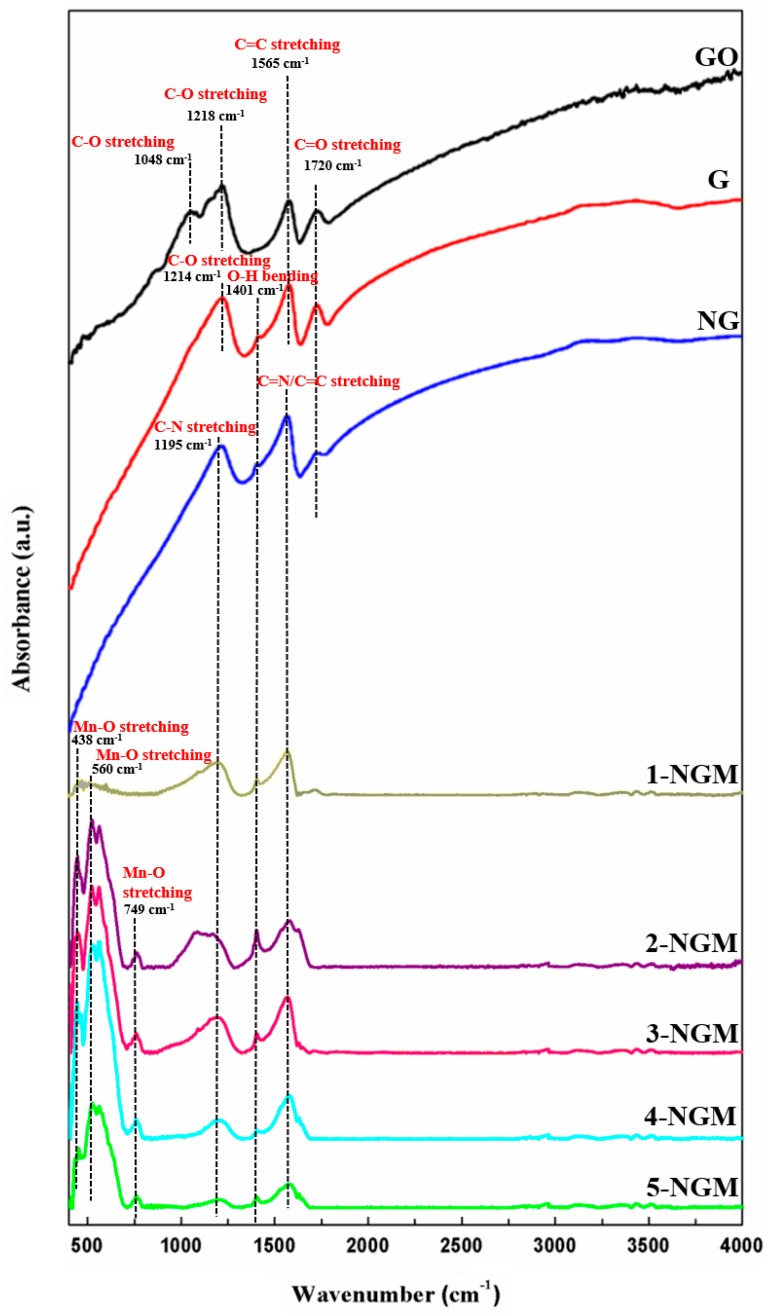
FTIR spectra of graphite oxide, G, NG, and x-NGM composites.

The functional group types on the surface of a material can be identified by the FTIR technique. Figure 4 shows the FTIR spectra of graphite oxide, G, NG, and x-NGM composites. For graphite oxide, the main absorption peaks are at 1084 cm^−1^ and 1218 cm^−1^, which are ascribed to the C-O stretchings. For G and NG, the two peaks at 1401 cm^−1^ and 1565 cm^−1^ are attributed to the O-H bending and C=C stretching, respectively. Graphite oxide, G, and NG all show the peak at 1720 cm^−1^, which is ascribed to the C=O stretching. Another peak for G at 1214 cm^−1^ can be assigned to the C-O stretching [62,63,64,65]. For the N-containing composites (NG and x-NGM), the two main absorption peaks are at 1195 cm^−1^ and 1565 cm^−1^, which are attributed to the C-N and C=N/C=C stretchings, respectively [66,67]. For the x-NGM composites, the two peaks at 438 cm^−1^ and 560 cm^−1^ can be ascribed to the Mn-O stretching [64,65,66,67]. One more peak for 2-NGM to 5-NGM is observed at 749 cm^−1^, which can also be attributed to the Mn-O stretching, and not be found in the composite without or with too less content of Mn (such as 1-NGM). The FTIR results mentioned above also contribute to confirming the successful preparation of the G, NG, and x-NGM composites.

**Figure 5:**
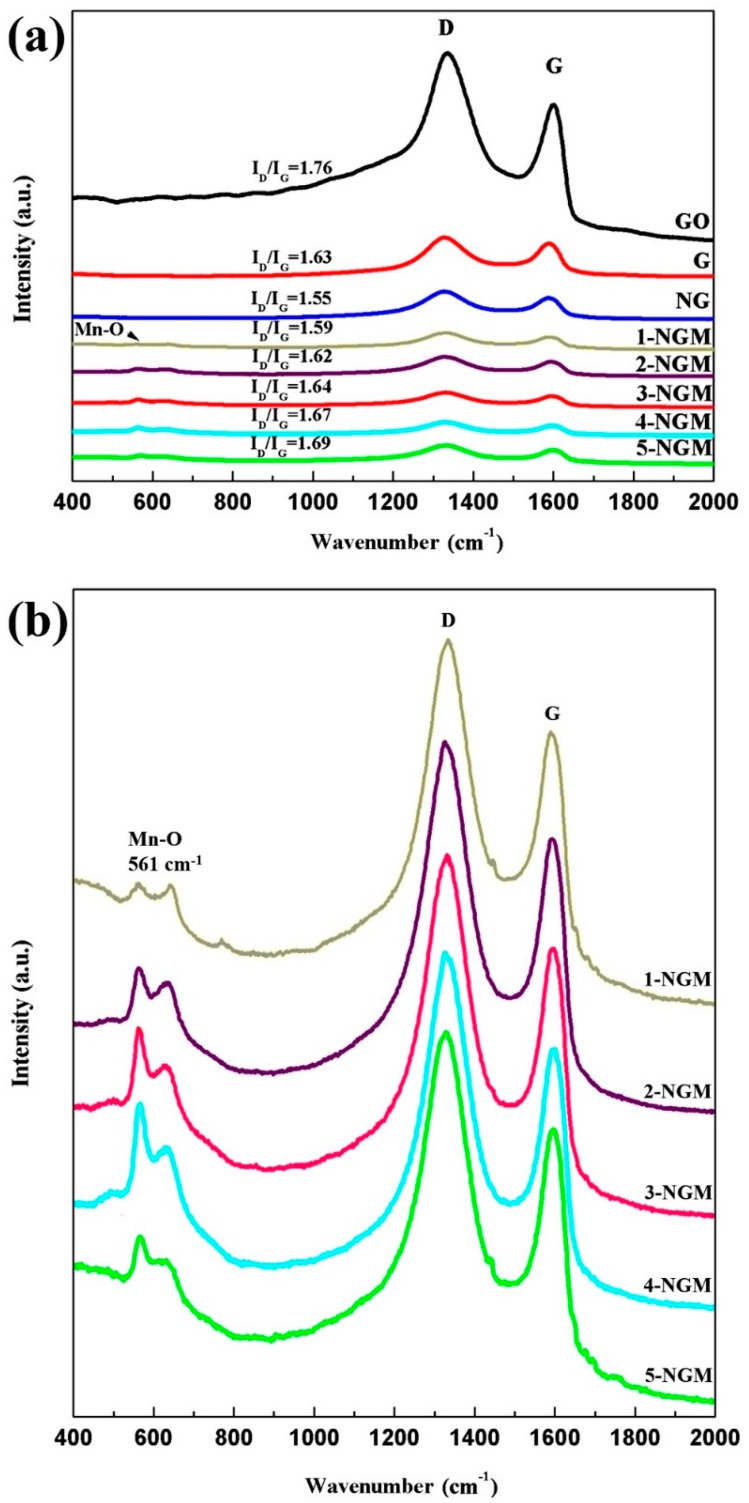
(**a**) Raman spectra of graphite oxide, G, NG, and x-NGM composites; (**b**) enlarged Raman spectra of x-NGM composites.

Raman spectroscopy is a widely used technique to examine the structures and electronic properties of G and its derivatives. Figure 5 shows the Raman spectra of graphite oxide, G, NG, and x-NGM composites, ranging from 400 cm^−1^ to 2000 cm^−1^. The two feature peaks at 1329 cm^−1^ and 1590 cm^−1^ are D and G bands, respectively [52,68,69]. The D band is due to the presence of disorders in sp^2^-hybridized carbon systems. It can be used to estimate the defect level and content of impurity in the G sheets. The G band is derived from the stretching of sp^2^-hybridized carbon-carbon bonds and highly sensitive to strain effects in the sp^2^ system within the G sheets. Furthermore, the intensity ratio of D and G bands, I_D_/I_G_, can be considered as a measure of the relative concentration of local defects or interferences, i.e., can be used to estimate the extent of sp^3^ graphite oxide converting to sp^2^ G [68,69]. Thus, an increment of the I_D_/I_G_ value implies an increase in the number of defects. From Figure 5a, the I_D_/I_G_ value of graphite oxide obtained before the hydrothermal process is calculated to be 1.73. However, those of the G, NG, and x-NGM composites drop to in between 1.55 to 1.69 after the hydrothermal process. It can be then deduced from the reduced I_D_/I_G_ values that the hydrothermal process could remove oxygen-containing functional groups and reduce graphite oxide to G successfully. This again helps to ascertain the presence of G in NG and x-NGM composites. Since the incident light wavelength of the Raman spectrometer influences excitation efficiency, the wavelength of 532 nm regarded as comparatively of less negative impact on enhancing the characteristic peak of Mn-O bonds was chosen to excite the x-NGM composites. However, as shown in Figure 5a, it still unavoidably resulted in weaker scattering intensity and thereby less distinct Mn-O characteristic peaks. After magnification, the peak at around 560 cm^−1^ attributed to the stretching vibration of Mn-O bonds [52] can be more clearly seen in Figure 5b, again confirming that the hydrothermal preparation of x-NGM composites was successful.

**Figure 6:**
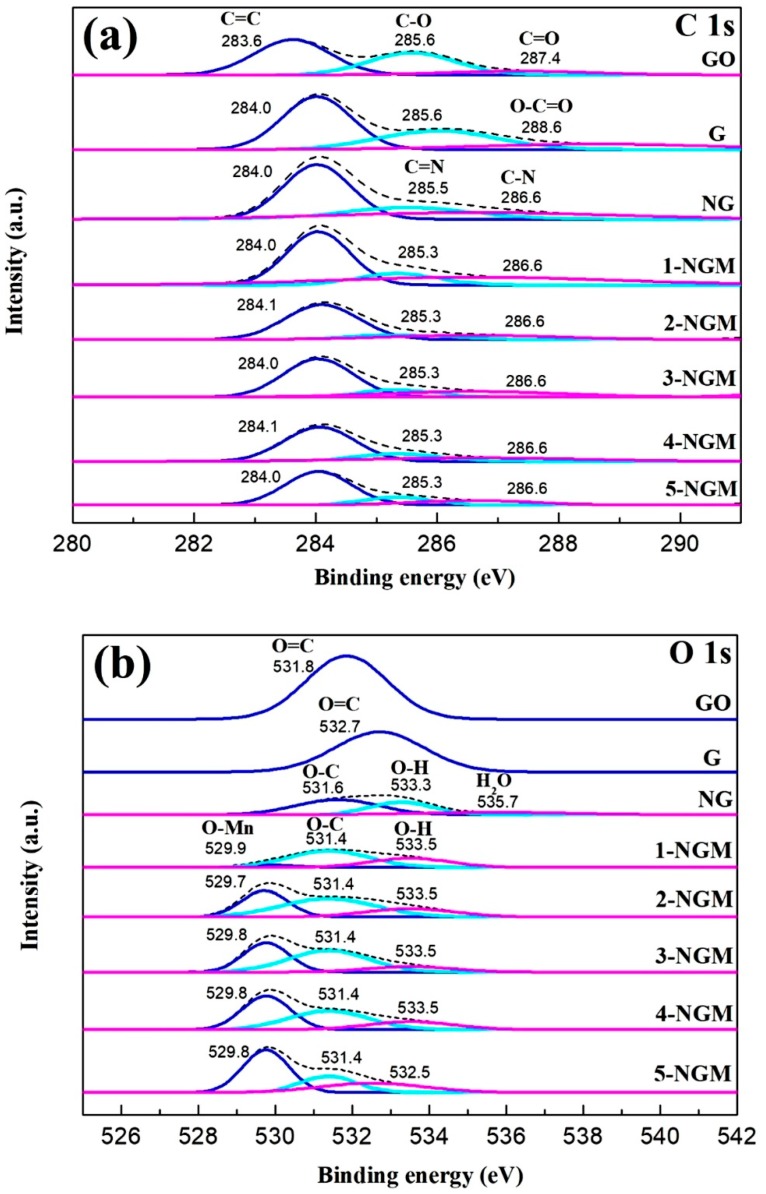

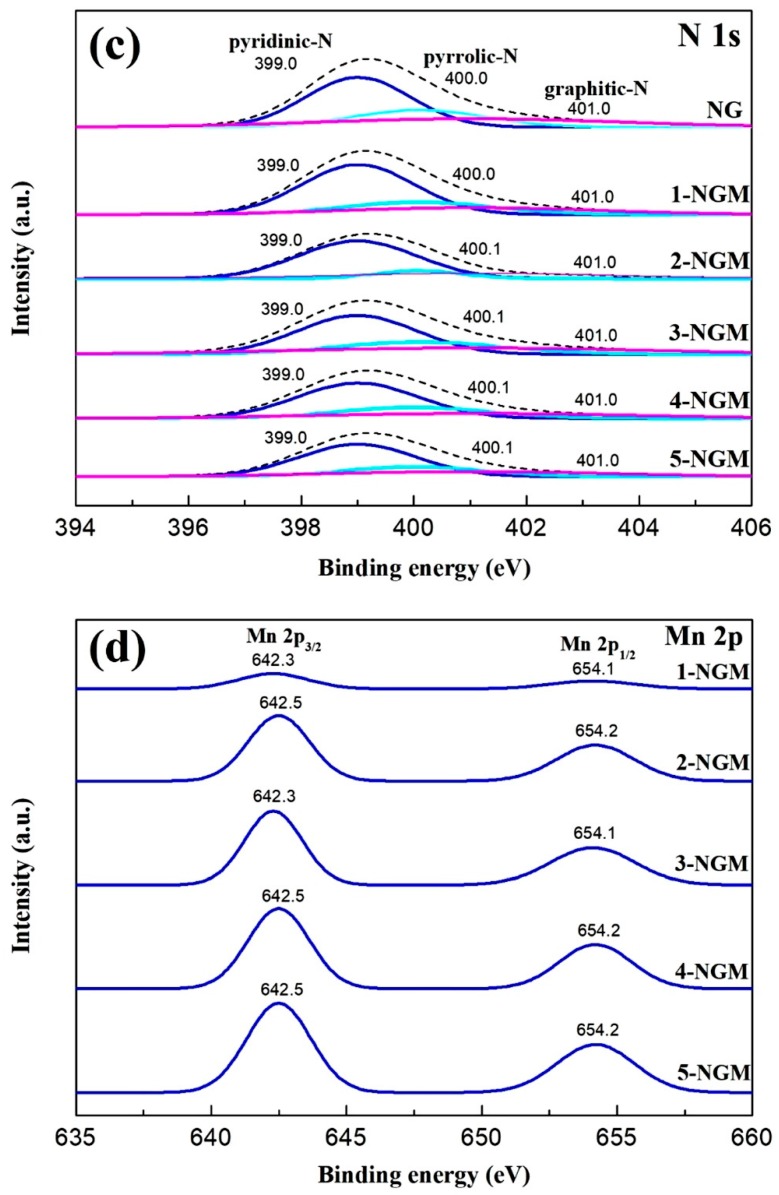
XPS spectra of graphite oxide, G, NG, and x-NGM composites: (**a**) C 1s, (**b**) O 1s, (**c**) N 1s, and (**d**) Mn 2p.

Figure 6 shows the XPS spectra of graphite oxide, G, NG, and x-NGM composites. The composition of a composite and chemical states of elements can be investigated by XPS. Figure 6a displays the C 1s spectra. For graphite oxide and G, they have a strong energy peak centered at approximately 283.8 eV, which is assigned to the C=C bonds (sp^2^-hybridized carbon atoms). Another weak peak with higher binding energy at 285.6 eV can be assigned to the C-O bonds (oxygenated carbon atoms) [62,63,70]. There is a weaker peak for graphite oxide at 287.4 eV, which is assigned to the C=O bonds. G shows an even weaker peak at 288.6 eV, which is assigned to the O-C=O bonds [52,62,63,70]. For NG and x-NGM composites, they also have energy peaks ascribed to the C=C bonds (symbol of the presence of G), centered at approximately 284.0 eV. When the containing of Mn in x-NGM to diminish the C=C bonds and sp^2^-hybridized carbon atoms, the peak intensity is found to be weakened. Another two weaker peaks of NG and x-NGM, centered at approximately 285.3 eV and 286.6 eV, can be assigned to the C=N and C-N bonds, respectively [62,63,69], which stand for another evidence for the existence of N element in the composites.

Figure 6b displays the O 1s spectra. Both graphite oxide and G show an energy peak ascribed to the C=O bonds at 531.8 eV and 532.7 eV, respectively. The three energy peaks of NG at 531.6 eV, 533.3 eV, and 535.7 eV, can be assigned to the bondings of O-C, O-H, and H_2_O, respectively [52,62,63]. The five x-NGM composites have similar O 1s spectra. They all exhibit the three energy peaks centered at approximately 529.8 eV, 531.4 eV, and 533.5 eV, which correspond to the Mn-O, C-O, and O-H bonds, respectively [52,62,63,69]. The increase in the content of Mn is found to enhance the intensity of the 529.8 eV energy peak. Figure 6c shows the N 1s spectra. All the six NG and x-NGM composites exhibit a strong peak at 399.0 eV and two weak peaks at 400.1 eV and 401.0 eV, which can be attributed to the three types of N-containing species on the surface: pyridinic-N, pyrrolic-N, and graphitic-N (quaternary N), respectively [52,70,71]. Again, the presence of the N element in the six active materials is confirmed. The fabrication of NG from G is demonstrated to be successful as well. Figure 6d shows the high-resolution Mn 2p spectra. All the five x-NGM composites exhibit the two peaks centered at approximately 642.3 eV and 654.1 eV, which are ascribed to the Mn 2p_3/2_ and Mn 2p_1/2_ spin-orbit splitting states, respectively. The separation of spin energy between the two peaks is 11.8 eV, indicating the oxidation state of Mn is +4. [52]. Moreover, the intensity of the two peaks is found to increase with an increased content of Mn. This not only confirms the presence of Mn in the x-NGM composites, but also demonstrates that the use of a hydrothermal method for preparation of the composites containing both NG and MnO_2_ was successful [52,62,63,72]. The XPS surveys have confirmed the presence of C, Mn, O, and N on the surface of the x-NGM composites.

**Figure 7:**
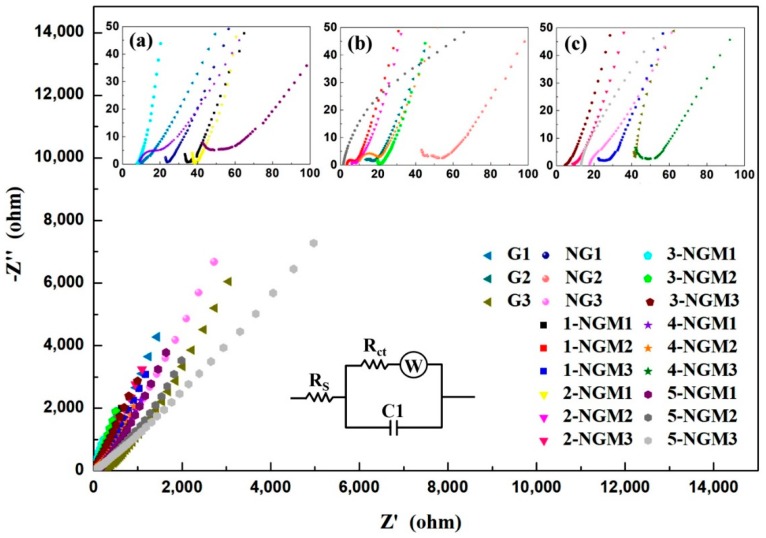
Nyquist plots of the 21 electrodes with Gy, NGy, and x-NGMy composites. Insets are enlargements when the mass loading is (**a**) 1 mg, (**b**) 2 mg, and (**c**) 3 mg.

EIS is a technique used for acquiring information of internal impedances in an electrochemical system. The electronic and ionic transports along the bulk and across the interface of active material in the electrode were thus investigated. The Nyquist plots of the 21 electrodes with different active materials are displayed in Figure 7, where the compressed semicircles at the high and medium frequency regions are related to the electronic transport resistance, a kinetic-controlled process. The line tail connecting the semicircle at the low-frequency region is associated with the ionic diffusion resistance, a thermodynamic-controlled process. The equivalent circuit for the EIS analysis is also depicted in Figure 7, which includes [73]: (1) the charge transfer impedance in the high-frequency region at the electrode/electrolyte interface (R_CT_), (2) the solution resistance (R_S_), which is the contact series resistance between the substrate and current collector, (3) Warburg impedance (W), which is the diffusion resistance of ions in the electrolyte and in relation to the slope of the line tail in the low-frequency region, (4) the electric double-layer capacitor (C_1_) [65,74,75,76]. The corresponding R_CT_ values obtained by simulation are listed in Table 1. By contrast, G1, NG1, 1-NGM1, 2-NGM1, 3-NGM1, 4-NGM1, and 5-NGM1 are found to exhibit smaller semicircles in the high-frequency region. Their R_CT_ values are 3.27 Ω, 2.22 Ω, 2.17 Ω, 1.28 Ω, 1.15 Ω, 8.06 Ω, and 9.29 Ω, respectively. When the mass loading of active materials on the substrate increases to 2 mg, the R_CT_ values of G2, NG2, 1-NGM2, 2-NGM2, 3-NGM2, 4-NGM2, 5-NGM2 are 3.52 Ω, 2.98 Ω, 2.49 Ω, 1.35 Ω, 1.23 Ω, 8.43 Ω, and 9.40 Ω, respectively. When the mass loading further increases to 3 mg, the R_CT_ values of G3, NG3, 1-NGM3, 2-NGM3, 3-NGM3, 4-NGM3, and 5-NGM3 are 12.83 Ω, 9.21 Ω, 8.42 Ω, 2.14 Ω, 1.70 Ω, 9.46 Ω, and 11.60 Ω, respectively. According to the significantly increased R_CT_ and larger semicircle in the high-frequency region, it can be then concluded that the preferred mass loading of active materials on the PI/graphite flexible substrate is 1 mg.

As shown in Table 1, after N was involved in the active materials to obtain NG composites, the R_CT_ value decreased from 3.27 Ω (G1) to 2.22 Ω (NG1). The interconnected NG component can effectively facilitate electronic transport. When N and MnO_2_ were simultaneously involved in the x-NGM composites, the R_CT_ value was further reduced from 2.22 Ω (NG1) to 1.15 Ω (3-NGM1). This indicates that the co-existence of NG and MnO_2_ in an active material could even more improve charge transfer. The diffusion/transport properties of electrolyte ions to the electrode surface can be examined by the linear response (slope) of the Nyquist plot in the low-frequency region. An increased slope usually illustrates a lower diffusion resistance, faster ionic transport, and thereby enhanced capacitive property [74,77]. As displayed in Figure 7, compared to those of G1 and NG1 electrodes, the Nyquist plot of the 1-NGM1 electrode has a larger slope in the low-frequency region, indicating its better ionic diffusion and higher conductivity since it contains NG and MnO_2_ simultaneously. The impact of the Mn content on R_CT_ was further investigated. Among the 15 electrodes with x-NGM active materials, the R_CT_ values for 1-NGM1, 2-NGM1, 3-NGM1, 4-NGM1, and 5-NGM1 are 2.17 Ω, 1.28 Ω, 1.15 Ω, 8.06 Ω, and 9.29 Ω, respectively. The 3-NGM1 electrode exhibits the smallest R_CT_, indicating its best charge transfer efficiency. Its Nyquist plot in the low-frequency region is almost vertical and shows the largest slope, representing the best ionic diffusion and capacitive properties of the 3-NGM1 electrode. It can be then deduced that the MnO_2_ nanowires growing on the NG surface provide more effective contacts between the electrode and electrolyte ions, and charge transfer is thus improved. However, excess Mn in the 4-NGM1 and 5-NGM1 electrodes causes increased R_CT_ values due to fewer contacts. Ion diffusion and charge transfer capacity are thereby suppressed, and a smaller slope of the Nyquist plot in the low-frequency region results. By the aforementioned results, it is confirmed that both the mass loading and content of Mn in an active material electrode affect conductivity. The best charge transfer efficiency can be obtained only when the mass loading is 1 mg and the content of Mn in x-NGM composites is optimized (x = 3).

**Figure 8:**
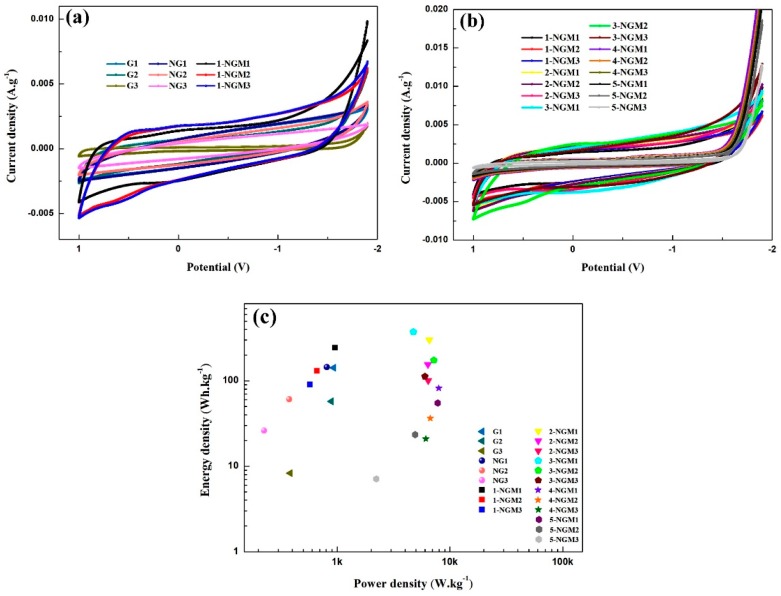
CV curves of the 21 electrodes with (**a**) Gy, NGy, 1-NGMy, and (**b**) x-NGMy composites. (**c**) Ragone plot obtained from the CV results.

The capacitive characteristic of an active material electrode can be evaluated by integrating the area inside a CV curve loop. Figure 8a,b shows the CV curves of the 21 electrodes with Gy, NGy, and x-NGMy composites, obtained at the scanning rate of 100 mV·s^−1^ with a fixed potential range of −2.9 V to 1.0 V. All x-NGMy composites show redox waves, indicating that the faradaic phenomena occurred during the charge/discharge process. No redox peaks are found for Gy and NGy composites. Their CV curves exhibit similar rectangular and symmetrical form, which is related to the characteristics of an electric double-layer capacitor. In Figure 8a, the CV curve loops of the 1-NGMy electrodes are the largest, indicating that they have the highest specific capacitances, followed by the NGy and then the Gy electrodes. Among them, the 1-NGM1 electrode has the largest area inside the loop, giving rise to a high specific capacitance of 416 F·g^−1^. Its energy and power densities obtained by calculations are 243.2 Wh·kg^−1^ and 959.9 W·kg^−1^, respectively. The capacitance enhancement can be ascribed to the synergistic effect of the higher conductivity by NG and the larger specific surface area by MnO_2_ nanostructures. Afterward, the content of Mn was tuned by using various KMnO_4_ concentrations. The impacts of mass loading on the capacitive property were also studied by using 1 mg, 2 mg, and 3 mg of active materials (y = 1, 2, and 3) for each x. From the CV curve loops of the x-NGMy electrodes in Figure 8b, the 3-NGM1 electrode shows the best capacitance performance with the highest specific capacitance of 638 F·g^−1^. Its energy and power densities are 372.7 Wh·kg^−1^ and 4731.1 W·kg^−1^, respectively. All the capacitance parameters acquired by calculations are listed in Table 2, which reveals that there was a most appropriate KMnO_4_ concentration, i.e., 26.72 mM when x = 3, to achieve an NGM composite with the optimum Mn content. Moreover, the increase of specific capacitance is more significant by pseudocapacitive MnO_2_ than NG.

By the interlaced nanowire structures of MnO_2_, the contact between the electrode surface and electrolyte ions and the use of active materials are both improved. There are more sites for electrochemical reactions to occur, and enhanced diffusion of ions in the electrolyte favorable for capacitive characteristic has resulted. The higher KMnO_4_ concentrations during the preparation process, 35.62 mM and 44.53 mM when x = 4 and 5, led to excess MnO_2_ in the NGM composites, which grew into nanorod structures detrimental to more contact between the electrode surface and electrolyte ions. Inferior ion diffusion and worse capacitive characteristics of the 4-NGMy and 5-NGMy electrodes are thereby caused, as shown in Figure 8b and Table 2. It can be also seen from Table 2 that the mass loading of active material on the flexible electrode is also critical. 1 mg has been demonstrated to be the most appropriate mass loading. 2 mg and 3 mg cause overloaded active materials and thus reduced specific capacitance, energy, and power densities. By plotting energy density vs. power density obtained from the CV results and calculated by Equations (3) and (5), a Ragone plot is achieved, as shown in Figure 8c, which again demonstrates the best electrochemical performance of 3-NGM1 among the 21 x-NGMy electrodes when x = 3 and the mass loading of 1 mg were used.

**Figure 9:**
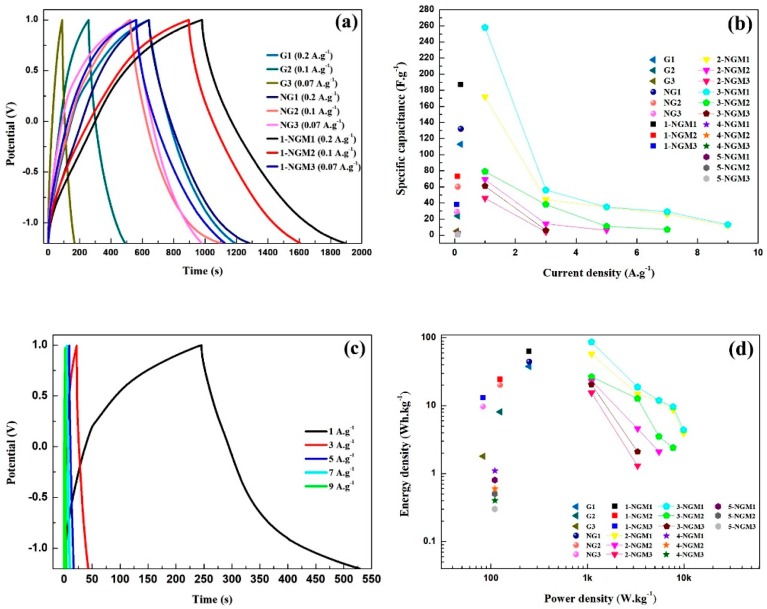
(**a**) GCD curves of the 9 electrodes with Gy, NGy, and 1-NGMy composites. (**b**) Plots of specific capacitance vs. current density for the 21 electrodes with Gy, NGy, and x-NGMy composites. (**c**) GCD curves of the 3-NGM1 electrode under the current densities of 1 A·g^−1^ to 9 A·g^−1^. (**d**) Ragone plot obtained from the GCD results.

The symmetry of charge and discharge curves can be investigated to understand capacitive behavior. Figure 9a shows the GCD curves of the 9 electrodes with Gy, NGy, and 1-NGMy composites, obtained by a fixed potential range of −1.2 V to 1.0 V under different current densities. Those of the 1-NGMy electrodes are slightly distorted from the ideal triangle shape because of the pseudocapacitive contribution from MnO_2_. The curvature implies that they are typical Faraday capacitance curves [78]. It is revealed that the 1-NGMy electrodes have the best capacitive characteristics, followed by the NGy electrodes, and the Gy electrodes are the worst. The 1-NGM1 electrode exhibits a longer charge and discharge time at a current density of 0.2 A·g^−1^, resulting in a specific capacitance of 188 F·g^−1^, and the corresponding energy and power densities are 63.1 Wh·kg^−1^ and 249.2 W·kg^−1^, respectively. Afterward, the impacts of the Mn content and mass loading on the capacitive parameters of the electrodes were also explored. The specific capacitances can be calculated from the GCD curves by Equation (2), as listed in Table 3. Figure 9b shows the plots of specific capacitance vs. current density for the 21 electrodes. The Gy, NGy, 1-NGMy, 4-NGMy, and 5-NGMy electrodes cannot endure the current densities larger than 1 A·g^−1^. The 2-NGM2 electrode can endure only the current densities of 1 A·g^−1^, 3 A·g^−1^, and 5 A·g^−1^. The 3-NGM2 electrode can endure the current densities of 1 A·g^−1^, 3 A·g^−1^, 5 A·g^−1^, and 7 A·g^−1^. The 2-NGM3 and 3-NGM3 electrodes can endure only the current densities of 1 A·g^−1^ and 3 A·g^−1^, whereas the 2-NGM1 and 3-NGM1 electrodes can endure all current densities. Among the 21 electrodes, the 3-NGM1 electrode exhibits the best endurance. Its energy and power densities are 86.7 Wh·kg^−1^ and 1100.0 W·kg^−1^, respectively. The above results have also confirmed the optimum conditions for the mass loading and content of Mn.

Since the 3-NGM1 electrode has shown the best sustainable ability to permit its higher charging capacity, it was selected to perform further GCD investigation by different current densities, are shown in Figure 9c. The rapid intercalation/deintercalation of metallic cations in an active material reveals the redox concerning the oxidation state transitions between Mn (III) and Mn (IV). It is inferred that at higher current densities, only the external surface of an active material is involved in charge/discharge, leading to insufficient redox and relatively lower specific capacitances. The charge/discharge time decreases along with a small number of electrolyte ions occupying the active sites. By contrast, at lower current densities, more internal and external active sites are involved, attaining more complete redox reactions and higher specific capacitances [79]. The increased charge/discharge time results from most electrolyte ions being anchored to the active sites at the interface. As displayed in Table 3, when the current density applied to the 3-NGM1 electrode increases from 1 A·g^−1^ to 9 A·g^−1^, the specific capacitance reduces from 258 F·g^−1^ to 13 F·g^−1^. The massive capacitance decay implies that the rate capability of the x-NGMy electrodes still needs considerable improvement. Moreover, it is perceived from Table 3 that the mass loading of active materials on the flexible electrode is very critical. 1 mg has been proven to be the optimum. Both 2 mg and 3 mg are overloads to cause lower conductivity and inferior capacitive parameters. For the Gy, NGy, and x-NGMy electrodes, their energy and power densities calculated from the GCD results are plotted as a Ragone plot, as shown in Figure 9d. Consistent with the EIS and CV results, the synergistic effect of NG with MnO_2_ is demonstrated again, to promote reversible redox reactions on the pseudocapacitive materials and play great impacts on the capacitance characteristics of the electrodes.

## 4. Conclusions

In this study, x-NGM composites consisting of NG and MnO_2_ with various Mn contents were fabricated by a low-cost hydrothermal method. By SEM, TEM, EDS mappings, XPS, FTIR and Raman spectra, the presence of NG and MnO_2_ was confirmed, and the successful preparation of the composites was demonstrated. The microstructure analysis by TEM manifested that the MnO_2_ in the x-NGM composites was a two-phase mixture of γ- and α-MnO_2_. According to the EIS results, the NG component was found to reduce R_CT_ effectively due to its good conductivity. The co-existence of NG and MnO_2_ in an active material led to a more reduced R_CT_ and further improved charge transfer. Among the 15 electrodes with x-NGM active materials, the 3-NGM1 electrode exhibited the smallest R_CT_, indicating its best charge transfer efficiency. Its Nyquist plot in the low-frequency region had the largest slope, implying a lower diffusion impedance, more rapid ionic diffusion, and enhanced capacitive property. Both the mass loading and content of Mn in an active material electrode were crucial. The best electrochemical performance was achieved when the mass loading of active materials on the PI/graphite flexible substrate was 1 mg and x = 3 to obtain the optimized Mn content in the x-NGM composites. Excess Mn caused decreased contacts between the electrode and electrolyte ions, leading to increased R_CT_, and suppressed ionic diffusion. Among the 21 electrodes with Gy, NGy, and x-NGMy composites, the 3-NGM1 electrode exhibited the best sustainable ability. However, its rate capability still required large improvement. After calculation of the CV results, it showed a high specific capacitance of 638 F·g^−1^, and the corresponding energy and power densities were 372.7 Wh·kg^−1^ and 4731.1 W·kg^−1^, respectively. The enhancement was ascribed to the synergistic effect of the higher conductivity by NG and the larger specific surface area by MnO_2_ nanostructures. Moreover, the increase of specific capacitance was found to be more significant by the pseudocapacitive MnO_2_ than NG.

## Figures and Tables

**Table 1:** R_CT_ values of the 21 electrodes with Gy, NGy, and x-NGMy composites obtained by EIS simulation.

Electrode	R_CT_ (Ω)	Electrode	R_CT_ (Ω)	Electrode	R_CT_ (Ω)
G1	3.27	1-NGM2	2.49	3-NGM3	1.70
G2	3.52	1-NGM3	8.42	4-NGM1	8.06
G3	12.83	2-NGM1	1.28	4-NGM2	8.43
NG1	2.22	2-NGM2	1.35	4-NGM3	9.46
NG2	2.98	2-NGM3	2.14	5-NGM1	9.29
NG3	9.21	3-NGM1	1.15	5-NGM2	9.40
1-NGM1	2.17	3-NGM2	1.23	5-NGM3	11.60

**Table 2:** Capacitance parameters obtained from the CV results of the 21 electrodes with Gy, NGy, and x-NGMy composites.

Electrode	Scan Rate (mV·s^−1^)	Specific Capacitance (F·g^−1^)	Energy Density (Wh·kg^−1^)	Power Density (W·kg^−1^)
G1	100	243	141.9	930.5
G2	100	99	57.5	889.0
G3	100	14	8.3	381.8
NG1	100	247	144.4	810.9
NG2	100	104	60.6	375.5
NG3	100	45	26.1	224.9
1-NGM1	100	416	243.2	959.9
1-NGM2	100	223	130.4	662.3
1-NGM3	100	154	90.1	572.9
2-NGM1	100	516	301.6	6556.8
2-NGM2	100	267	155.7	6370.1
2-NGM3	100	173	100.7	6407.3
3-NGM1	100	638	372.7	4731.1
3-NGM2	100	298	174.2	7208.0
3-NGM3	100	192	112.0	6001.7
4-NGM1	100	141	82.0	7959.5
4-NGM2	100	62	36.4	6689.0
4-NGM3	100	36	20.9	6105.0
5-NGM1	100	94	55.0	7799.2
5-NGM2	100	40	23.4	4918.4
5-NGM3	100	12	7.1	2227.0

**Table 3:** Capacitance parameters obtained from the GCD results of the 21 electrodes with Gy, NGy, and x-NGMy composites by different current densities.

Electrode	Current Density (A·g^−1^)	Specific Capacitance (F·g^−1^)	Energy Density (Wh·kg^−1^)	Power Density (W·kg^−1^)
G1	0.2	113	38.0	249.2
G2	0.1	24	8.1	124.6
G3	0.07	5	1.8	83.1
NG1	0.2	132	44.4	249.2
NG2	0.1	60	20.1	124.6
NG3	0.07	29	9.7	83.1
1-NGM1	0.2	188	63.1	249.2
1-NGM2	0.1	73	24.5	124.6
1-NGM3	0.07	39	13.1	83.1
2-NGM1	1	172	57.8	1100.0
2-NGM2	1	69	23.1	1100.0
2-NGM3	1	46	15.4	1100.0
3-NGM1	1	258	86.7	1100.0
3-NGM2	1	79	26.6	1100.0
3-NGM3	1	61	20.5	1100.0
2-NGM1	3	44	14.7	1100.0
2-NGM2	3	14	4.6	3300.0
2-NGM3	3	4	1.3	3300.0
3-NGM1	3	56	18.8	3300.0
3-NGM2	3	38	12.7	3300.0
3-NGM3	3	6	2.1	3300.0
2-NGM1	5	35	11.8	5500.0
2-NGM2	5	6	2.1	5500.0
3-NGM1	5	36	11.9	5500.0
3-NGM2	5	11	3.5	5500.0
2-NGM1	7	26	8.6	7700.0
3-NGM1	7	29	9.6	7700.0
3-NGM2	7	7	2.4	7700.0
2-NGM1	9	12	3.9	9900.0
3-NGM1	9	13	4.4	9900.0
4-NGM1	0.1	3	1.1	110.0
4-NGM2	0.1	2	0.6	110.0
4-NGM3	0.1	1	0.4	110.0
5-NGM1	0.1	2	0.8	110.0
5-NGM2	0.1	1.6	0.5	110.0
5-NGM3	0.1	1	0.3	110.0
